# Measurement of tissue cortisol levels in patients with severe burns: a preliminary investigation

**DOI:** 10.1186/cc8184

**Published:** 2009-11-27

**Authors:** Jeremy Cohen, Renae Deans, Andrew Dalley, Jeff Lipman, Michael S Roberts, Bala Venkatesh

**Affiliations:** 1 Burns Trauma and Critical Care Research Centre, University of Queensland, Butterfield St, Herston 4006, Australia; 2 Therapeutic Research Unit, University of Queensland, Princess Alexandra Hospital, Ipswich Rd, Woolloongabba, Queensland 4102, Australia; 3 Intensive Care Unit, Princess Alexandra Hospital and Wesley Hospitals, University of Queensland, Ipswich Road, 4102 Auchenflower, Australia

## Abstract

**Introduction:**

The assessment of adrenal function in critically ill patients is problematic, and there is evidence to suggest that measurement of tissue glucocorticoid activity may be more useful than estimation of plasma cortisol concentrations. Interstitial cortisol concentrations of cortisol represent the available pool of glucocorticoids able to enter the cell and bind to the glucocorticoid receptor. However the concentrations of plasma cortisol may not accurately reflect interstitial concentrations. We elected to perform a preliminary study into the feasibility of measuring interstitial cortisol by microdialysis, and to investigate the relationship between total plasma cortisol, free plasma cortisol and interstitial cortisol in patients with severe burns.

**Methods:**

A prospective observational study carried out in a tertiary intensive care unit. Ten adult patients with a mean total burn surface area of 48% were studied. Interstitial cortisol was measured by microdialysis from patient-matched burnt and non-burnt tissue and compared with that of 3 healthy volunteers. Plasma sampling for estimations of total and free cortisol concentrations was performed concurrently.

**Results:**

In the burn patients, mean total plasma and free plasma cortisol concentrations were 8.8 +/- 3.9, and 1.7 +/- 1.1 mcg/dL, (p < 0.001), respectively. Mean subcutaneous microdialysis cortisol concentrations in the burn and non-burn tissue were 0.80 +/- 0.31 vs 0.74 +/- 0.41 mcg/dL (p = 0.8), respectively, and were significantly elevated over the mean subcutaneous microdialysis cortisol concentrations in the healthy volunteers. There was no significant correlation between total plasma or free plasma and microdialysis cortisol concentrations. Plasma free cortisol was better correlated with total burn surface area than total cortisol.

**Conclusions:**

In this preliminary study, interstitial cortisol concentrations measured by microdialysis in burnt and non-burnt skin from patients with severe thermal injury are significantly elevated over those from healthy volunteers. Plasma estimations of cortisol do not correlate with the microdialysis levels, raising the possibility that plasma cortisol may be an unreliable guide to tissue cortisol activity.

## Introduction

The severely burned patient suffers from a rapidly changing pathophysiology in the immediate post-burn period characterized by wound inflammation, cardiopulmonary instability, systemic inflammatory response syndrome and metabolic derangement. One of the integral components of this stress response is the activation of the adrenal axis resulting in an exaggerated output of cortisol. A number of studies have demonstrated increases in total plasma cortisol and adrenocorticotrophic hormone (ACTH) concentrations in the days following thermal injury [1-3]. Urinary free cortisol levels have also been shown to be increased after burns for up to 100 days [4]. All of these changes would support the concept of an exaggerated adrenal response.

However, attempting to characterise the sufficiency of the adrenal response in this patient population has been problematic. Patients with burns pose specific problems with respect to the interpretation of adrenal function tests. The predominant focus of previous investigations has been total plasma cortisol (TC), yet it is the unbound, free cortisol that is the active fraction [5]. Cortisol binding globulin (CBG) levels are known to show significant variation following thermal injury and this will therefore impact on the levels of physiologically active cortisol [6]. Furthermore, total cortisol levels have been shown to be subject to significant hourly variability and inter assay variation [7,8]. Additionally, interpretation of stimulation tests in the setting of the severe pre-existing stress of a burn injury is difficult, because there is evidence that circulating endogenous ACTH levels will influence the cortisol response to exogenous ACTH [9].

### Relevance of interstitial cortisol measurements

Given the above difficulties, more recent investigation of adrenal function in the critically ill has examined the role of plasma free cortisol (PFC) [5,10] and tissue cortisol activity [11]. PFC is the bioactive fraction and is a critical determinant of tissue cortisol. However, plasma values are not the only determinant of interest. Free cortisol exerts its activity by passing through the cell membrane and binding to the cytosolic glucocorticoid receptor. Due to their lipophilic nature glucocorticoids passively diffuse through plasma membranes [12] and thus it is the free cortisol concentration in the interstitial fluid that is one of the principal determinants of the available glucocorticoid pool for receptor binding. Cortisol concentrations in plasma and interstitial fluid may not necessarily run in parallel and blood plasma to interstitial fluid exchange may be often compound specific. For example, we have shown that there is a significant dissociation between plasma and interstitial concentrations of antibiotics [13].

Microdialysis is an *in vivo *sampling technique for measuring endogenous and exogenous solutes in the extracellular space of tissue. A small probe equipped with a semi-permeable hollow fibre is inserted superficially into the dermis, and perfused with a solution that forms an equilibrium with diffusible molecules in the immediate surroundings [14]. Microdialysis techniques have recently been used to investigate interstitial cortisol concentrations (which are largely free) [15], thus allowing comparison with plasma values. Although routine measurement of tissue hormone concentrations may not be practical in the clinical setting, the assessment of a relation between plasma and interstitial concentrations may allow us to develop predictive models for tissue cortisol concentrations from plasma measurements.

The aims of this pilot study were: to examine the practicality and feasibility of using microdialysis techniques to estimate interstitial cortisol concentrations in patients with severe burns; and to examine the relation between circulating TC and PFC levels and interstitial cortisol.

## Materials and methods

### Study design

The plasma and microdialysis data for this study were obtained in conjunction with a separate study investigating antibiotic pharmacokinetics [13].

A burn site- and patient-matched paired comparison of burnt and non-burnt tissue cortisol microdialysate levels was conducted together with a non-paired comparison of microdialysate levels from non-burnt tissue sites in burn patients and healthy volunteers. Corresponding unbound plasma cortisol concentrations were obtained simultaneously.

### Ethical review

The protocol received approval from the Royal Brisbane Hospital and University of Queensland Human Research Ethics Committees. Written informed consent was obtained from the legal guardians of enrolled patients and from the healthy volunteers.

### Patient and volunteer enrolment

Ten adult patients with a mean ± standard deviation (SD) age of 32 ± 11 years and total burn surface area (TBSA) of 48 ± 15% were enrolled in the study. The patients were admitted to the Royal Brisbane & Women's Hospital intensive care unit between February 2005 and February 2006 and received eschar debridement and grafting surgery within the first few days post-injury, during which time the studies were conducted. Exclusion criteria included age younger than 18 years, existing bacterial infection and known infection with hepatitis A, B or C or HIV. Patients were resuscitated during the burn shock phase using the Parkland formula adjusted to patients' requirements [16]. No patients had been on chronic steroid therapy prior to enrollment or received etomidate or hydrocortisone during the period of the study. Inotropic or vasopressor support was instituted at the treating clinicians' discretion.

Three volunteers with a mean ± SD age of 35 ± 5 years were recruited exclusively from within the research group associated with the study. Exclusion criteria included age younger than 18 years or poor health as assessed by a medical practitioner.

### Burn patient and healthy volunteer study protocols

Patient studies were conducted during debridement and grafting procedures within five days of trauma (mean post-trauma delay before grafting: 2.2 ± 1.2 days; mean surgery duration: 5.7 ± 1.9 hours). Burn patient microdialysis sites were selected for anticipated ease of access during debridement surgery in body areas that were not expected to be required as skin graft donor sites, and were not scheduled for eschar debridement at this procedure. Full thickness burn sites and adjacent non-burnt skin areas in the neck/shoulder and groin/thigh areas were used. After insertion, probes were held in place with a surgical stitch, and were then covered with protective sterile gauze and stapled to avoid dislodgment during the debridement procedure in the operating theatre. The microdialysis site for volunteers was the volar forearm. Patient and volunteer microdialysis sites were anaesthetised with 1% lignocaine (Xylocaine^®^, AstraZeneca, Luton, UK) before probe insertion. The probe was held in place with Tegaderm™ (3 M Health Care, St Paul, MN, USA). CMA 60 microdialysis probes (CMA, Stockholm, Sweden) were perfused with aseptically prepared 0.9% saline containing 2 mg/L cefazolin at a flow rate of 1.6 μL per minute from a 1 mL syringe using a Graseby^® ^MS16 24 h syringe driver (Smiths Group plc, London, UK). Microdialysis probes were perfused for up to 30 minutes prior to insertion to remove the preservative buffer. Probe perfusate was collected into sterile CMA collection vials, transferred to reduced volume 300 μL polypropylene autosampler vials (AH0-7777, Phenomenex, Torrance, CA, USA) and stored at -20°C. Cortisol concentrations were determined in 20 minute microdialysate collections that were taken 5.3 ± 2.1 hours after commencement of surgery.

### Cortisol analysis

Cortisol analysis was by ELISA using a commercial kit (Cortisol assay # KGE008, R&D Systems Inc, Minneapolis, MN, USA) in exact accordance with the manufacturer's instructions. Briefly, the assay employs competitive ELISA principles in a 96-well plate and has a horseradish peroxidise/3,3',5,5'-tetramethylbenzidine endpoint read at 450 nm λ with wavelength correction at 540 nm λ. We used a Paradigm™ Detection Platform and Multimode Analysis Software version 3.1.0.1 (Beckman Coulter Inc, Fullerton, CA, USA) for quantification.

In our study the ELISA gave an inter-assay coefficient of variation (CV) of 6.36% (for 2.5 ng/mL on five occasions) and a dynamic range of 0.312 to 10 ng/mL cortisol. A linear ELISA response to cortisol dilution with saline was demonstrated.

### Sample dilution

All samples required dilution prior to ELISA analysis to ensure that their cortisol values could be read from the standard curve. Unprocessed plasma samples were all diluted 1/20 in accordance with the manufacturer's instructions. Ultracentrifuged plasma samples were diluted 1/5 and 1/2. For microdialysis samples a dilution factor of 1/3 was optimal for 68% of analyses. Additional dilution factors were required for six microdialysis samples.

### Unbound plasma cortisol determination

Blood was sampled into heparinised vacutainers^® ^(BD, Beckton-Dickinson, Rutherford NJ, USA) from an indwelling arterial cannula for patients and from an indwelling venous cannula for volunteers, processed and stored at -20°C. Patient plasma sample times differed to microdialysis sample times by 0.7 ± 0.6 hours. Ultracentrifugation methods were used to isolate unbound plasma cortisol fractions. Briefly, 500 μL of plasma was incubated at 37°C for 30 minutes and ultracentrifuged at 12,000 g for 20 minutes through 30 KDa nominal cut-off membrane devices (Amicon^® ^YM30, Millopore Corporation, Billerica, MA, USA) to give a filtrate yield of approximately 25% original volume that was analysed by cortisol ELISA.

### Statistical analysis

Continuous, normally distributed variables were summarised as mean ± SD. Differences in cortisol concentrations between groups were analysed using independent t-tests. The degree of association between variables was assessed using Spearman's correlation coefficient. Statistical significance was taken at a level of 5%.

## Results

Thirteen subjects were enrolled into the study; 10 burns patients and three healthy volunteers. Demographic data for the burns patients are presented in Table 1. Of these patients, 80% were male, with an average age of 32 ± 11 years and TBSA of 48 ± 15%.

**Table 1:** Patient demographics

Patient number	APACHE II	Burn area (%)
1	14	53

2	15	35

3	15	45

4	9	30

5	13	28

6	9	45

7	13	70

8	11	45

9	8	40

10	13	65

APACHE = acute physiology and chronic health evaluation.

Plasma and microdialysis values are presented in Table 2. Two plasma and one microdialysis sample from patients six and nine were unsuitable for analysis.

**Table 2:** Plasma and tissue cortisol measurements

Patient number	Total plasma cortisol (μg/dl)	Free plasma cortisol (μg/dl)	Microdialysis cortisol burn tissue (μg/dl)	Microdialysis cortisol non-burn tissue (μg/dl)	Requiring vasopressors
1	10.7	3.4	0.4	0.3	No

2	3.0	0.3	0.5	0.1	No

3	12.0	2.4	0.8	1.0	No

4	2.2	0.1	0.5	0.5	Yes

5	11.0	1.4	1.2	0.7	Yes

6			1.3	1.1	No

7	7.2	1.3	1.2	1.6	No

8	11.1	1.9	0.6	0.8	Yes

9			4.5		Yes

10	13.2	3.1	1.3	0.8	Yes

**Volunteer**	**Microdialysis cortisol (μg/dl)**				
				
1	0.3				
				
2	0.2				
				
3	0.1				

Mean TC and PFC concentrations were 8.8 ± 3.9 and 1.7 ± 1.1 μg/dL (*P *< 0.001), respectively. Mean microdialysis cortisol concentrations in the burn (MDB) and non-burn tissue (MDNB) were 0.80 ± 0.31 vs 0.74 ± 0.41 μg/dL (*P *= 0.8), respectively.

TC was significantly elevated with respect to both the MDB and MDNB concentrations (*P *< 0.001); however, PFC was significantly elevated over MDNB cortisol (1.7 ± 1.1 vs 0.74 ± 0.41; *P *= 0.05) but not MDB (1.7 ± 1.1 vs 0.80 ± 0.31, *P *= 0.06).

Compared with the healthy controls both the MBD and MBNB cortisol concentrations were significantly elevated; 0.80 ± 0.31 and 0.74 ± 0.41 vs 0.20 ± 0.05 μg/dL (*P *= 0.003, *P *= 0.004), respectively.

### Correlative analysis

We examined the correlation between TC and PFC concentrations, MCB and MDNB concentrations, and TBSA. Overall, there were no statistically significant correlations.

**Figure 1:**
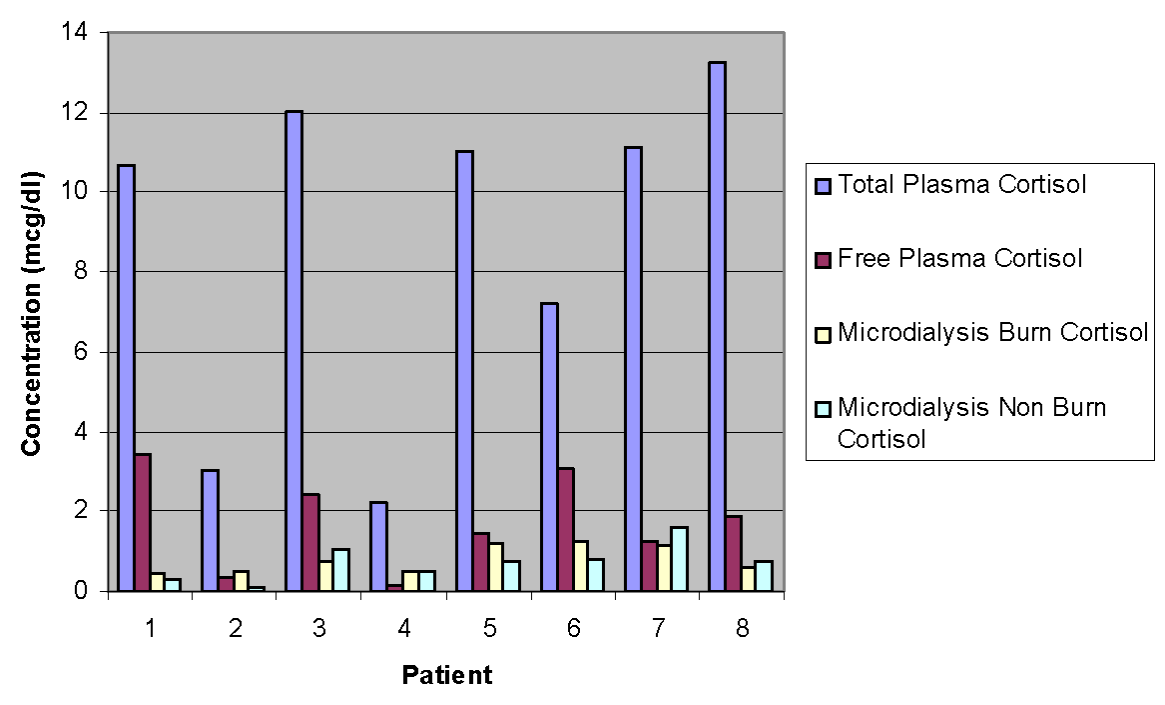
Plasma and interstitial cortisol values

TC was well correlated with PFC (r = 0.59) but less well correlated with MDB (r = 0.3). Similarly, the correlation between PFC and MDB was poor (r = 0.2). This poor correlation was reflected in the observation that 20% of the MDB concentrations were higher than the corresponding plasma PFC values. TC and PFC, MDB and MDNB values are presented in Figure 1.

TBSA was correlated best with the plasma PFC concentration (r = 0.54), and less so with the TC (r = 0.46) and MBD (r = 0.35). However, there was a better correlation between MDNB and TBSA (r = 0.54).

## Discussion

To the best of our knowledge, this is the first study to examine interstitial cortisol concentrations in a critically ill population suffering from severe burns. We have demonstrated the feasibility of measuring interstitial cortisol concentrations in patients with burns. Our preliminary data also indicate that interstitial cortisol levels are significantly elevated over normal controls, and that there is no significant correlation between free cortisol and microdialysis cortisol concentrations taken from either burned or non-burned tissue. As can be seen from Figure 1 in several cases microdialysis concentrations were higher than those of plasma.

Glucocorticoids (GC) are known to play an essential role in the response to critical illness. Although absolute adrenal insufficiency is a well recognised, but rare, clinical entity, relative adrenal insufficiency (or critical illness-related corticosteroid insufficiency) is a less well-recognised phenomenon, in which it is postulated that there may be a blunted adrenal response to stress or a tissue resistance to GC action. Identification of patients with this syndrome is of clinical importance, because they may potentially benefit from cortisol supplementation in the form of hydrocortisone; however, results from clinical trials of hydrocortisone in the setting of septic shock have been inconclusive [17,18], which may be in part due to an inability to effectively measure adrenal function in this patient population. Previous diagnostic criteria have been primarily focused on the measurement of TC values, taken either as a random baseline or as part of a stimulation test in response to synthetic ACTH. However, TC measurement has a number of drawbacks including: poor correlation with the active, free hormone concentrations; poor reproducibility; significant hourly fluctuations; and significant intra-assay variations [5,7,8,10,19]. Recognition of these limitations has led to the recommendations in the latest surviving sepsis guidelines that plasma cortisol values should not be used for the identification of patients with potential adrenal insufficiency [20].

Previous studies in burns patients have demonstrated elevations of TC, but these have been highly variable ranging from average concentrations of 12.4 to 32 μg/dL [21,22]. The relation between TBSA and TC is also unclear, because some investigators have been able to demonstrate a correlation [2], while others have not [21].

Investigations into PFC levels in burns have been more limited [6,23] but likewise suggest that PFC levels are initially increased after burn injury.

In our study the TC levels were surprisingly low, (8.8 ± 3.9 μg/dL) for the degree of stress and indeed fall into the range observed in healthy volunteers [5]. However, TC values in this range have been reported in other studies [11,24,25]. In contrast, the PFC values were elevated over the normal reference range [5]. However, the PFC concentrations in burns patients reported by Bernier and colleagues [6], range between 12 and 16 μg/dL, which are significantly higher than those seen in our patients, and in those reported in septic shock [5,10]. There are a number of possible reasons for this discrepancy. TC values in burns patients may be influenced by numerous factors, including time of sampling, TBSA, CBG levels, effect of resuscitation, and general anaesthesia. It is noteworthy that our samples were taken on average several days after the injury, and during surgical debridement. General anaesthesia, time after burn injury, blood transfusion in the setting of surgery, and differing resuscitation protocols may all have significant effects on our measured cortisol values. In addition, our results indicated that PFC was better correlated with TBSA than TC. To our knowledge this observation has not been made before, and is consistent with studies in sepsis indicating that PFC is more closely correlated with sickness severity than TC [10].

A potentially more accurate estimation of adrenal axis function may come from examining tissue GC activity. The interstitial cortisol concentration represents the available GC pool, which is able to enter the cell and bind to the GC receptor. As such, it is therefore a more accurate marker of tissue cortisol activity than plasma concentrations. However, the reference range for interstitial cortisol in the critically ill patient is unknown. It has historically been assumed that TC concentrations determine PFC concentrations which in turn determine interstitial cortisol concentrations; the so called 'cortisol cascade'. We have demonstrated that interstitial cortisol concentrations are significantly elevated in both burnt and non-burnt tissue from patients with severe thermal injury, and that the correlation between interstitial and plasma concentrations of cortisol is poor. It is particularly noteworthy that in 20% of cases, microdialysis cortisol concentrations from burned tissue were higher than the corresponding plasma values.

There are a number of possible explanations for these findings, including generation of interstitial free cortisol, diffusion of intracellular cortisol, and local pharmacokinetic factors.

Cortisol can be cleaved from cortisol binding globulin by the actions of neutrophil elastase, an enzyme released from polymorphonuclear leukocytes at the site of inflammation [26]. The extensive inflammatory response engendered by severe burn injury may therefore lead to increased interstitial cortisol concentrations via this mechanism. Additionally, intracellular cortisol, generated from cortisone secondary to the activity of 11 betahydroxysteroid dehydrogenase 1 enzyme, can diffuse into the interstitium [15], thus contributing to the interstitial pool of free cortisol.

Other factors may influence interstitial cortisol concentrations. These include interstitial fluid volume, capillary 'leakage' and peripheral tissue perfusion, all of which are likely to be significantly abnormal in patients with severe burns. Extensive tissue oedema is characteristic of severe thermal injury, and appears to be related to increased capillary permeability, vigorous fluid resuscitation, and changes in interstitial fluid pressure [27]. Increased capillary permeability has been documented to increase in both burned and non-burned tissue following thermal injury [28], which may explain the lack of difference in MDB and MDNB cortisol concentrations in our group. Vasopressor use is also frequent in the management of serious burns, and the subsequent vasoconstriction can reduce tissue perfusion, thus potentially reducing cortisol clearance. Of note was that 50% of our subjects were receiving noradrenaline infusions at the time of enrolment.

Similar pathophysiological changes to those of burns can be observed in subjects suffering from trauma or severe sepsis, and studies in these groups have demonstrated significant variations in the interstitial concentrations of antibiotics compared with healthy controls [29,30].

Our study has a number of limitations, primarily it has a limited sample size. We did not perform ACTH testing, because the rapidly changing physiology of the operative setting would make the results difficult to interpret. Moreover, as noted earlier, stimulation testing in critically ill patients is subject to a number of errors. We are also unable to comment as to whether the divergence between plasma and interstitial values we have demonstrated in skin would be replicated in other tissues. However, our intent was that of hypothesis generation into cortisol kinetics in the critically ill patient as a platform for planning future trials.

## Conclusions

In this preliminary study, we have shown that microdialysis techniques can be used to estimate interstitial cortisol concentrations in critically ill patients. Plasma estimations of cortisol do not correlate with the microdialysis levels raising the possibility that plasma cortisol may be an unreliable guide to tissue cortisol activity.

## Key messages

• Interstitial cortisol concentrations can be measured by microdialysis.

• In this pilot study interstitial cortisol concentrations in patients with burns were elevated with respect to controls, and poorly correlated with plasma values.

## Abbreviations

ACTH: adrenocorticotrophic hormone; CBG: cortisol binding globulin; ELISA: enzyme linked immunosorbent assay; GC: glucocorticoids; MDB: microdialysis concentrations from burn tissue; MDNB: microdialysis concentrations from non-burned tissue; PFC: plasma free cortisol; SD: standard deviation; TBSA: total burn surface area; TC: total cortisol.

## Competing interests

The authors declare that they have no competing interests.

## Authors' contributions

JC contributed to the concept and design of the study and drafted the manuscript. RD carried out patient enrolment and coordinated specimen collection. AD assisted with specimen collection and performed the assays. JL assisted with study concept and design and assisted with revision of the manuscript. MR assisted with study concept and design. BV contributed to the design of the study and assisted with draft and revision of the manuscript.
